# Physical and Chemical Property Changes, Cooked-Off Flavor Formation, and Its Alleviation During Storage of Green Tea Beverages

**DOI:** 10.3390/foods15101656

**Published:** 2026-05-09

**Authors:** Ning Chi, Yu-Rong Hu, Zhou-Tao Fang, Cun-Yu Li, Hong-Zhiyuan Yang, Shan-Shan Wu, Xin-Qiang Zheng, Jian-Hui Ye, Yue-Rong Liang, Ding-Wu Zhang, Jian-Liang Lu

**Affiliations:** 1 Tea Research Institute, Zhejiang University, Hangzhou 310058, China; chn202308@163.com (N.C.); ztfang@zju.edu.cn (Z.-T.F.); 12016067@zju.edu.cn (C.-Y.L.); hongzyyang@163.com (H.-Z.Y.); wss923@163.com (S.-S.W.); xqzheng@zju.edu.cn (X.-Q.Z.); jianhuiye@zju.edu.cn (J.-H.Y.); yrliang@zju.edu.cn (Y.-R.L.); 2 KangShi (Shanghai) Food Science and Technology Co., Ltd., Shanghai 200120, China; huyurong@masterkong.com.cn; 3 Shaoxing Jianming Tea Industry Co., Ltd., Hangzhou 310058, China; 4 Market Supervision and Administration Bureau of Huaxi District, Guiyang 550029, China

**Keywords:** green tea beverage, storage treatment, sensory quality, quality stability, cooked-off flavor

## Abstract

Quality deterioration, characterized by browning and cooked-off flavor (COF) development, often occurs in green tea beverages (GTBs) during shelf life. Changes in sensory quality and physical and chemical properties, as well as COF formation and its alleviation under different storage conditions, were comprehensively investigated in beverages prepared from five green teas and one jasmine-scented green tea. As the storage temperature increased and the storage duration extended, all GTBs exhibited a noticeable decrease in total sensory score (TSS), along with remarkable increases in total color difference (ΔE) and COF. Contents of total polyphenols declined markedly during storage of GTBs, while levels of caffeine and free amino acids changed insignificantly. Among the nine volatiles negatively correlated with TSS and positively correlated with COF, linalool, geraniol, 1-octanol, and 3-methylbutanal were identified as key COF contributors due to their high relative odor activity values. These results indicate that quality deterioration of GTBs during storage is closely associated with polyphenol loss, accumulation of COF-related components, and shifts in volatile composition and ratios. Notably, beverages prepared from jasmine-scented tea exhibited higher sensory scores and slower quality deterioration during storage than those prepared from unscented tea. This study identifies the key volatile contributors to COF and demonstrates that jasmine scenting effectively mitigates quality deterioration, offering a practical strategy for extending the shelf life of GTBs.

## 1. Introduction

Tea beverages, one of the most popular non-alcoholic drinks globally, are typically produced via extraction, clarification, sterilization, and drying [1,2]. Based on their final form, tea beverages can be divided into liquid products (e.g., concentrated tea juice and ready-to-drink (RTD) tea) and solid products (e.g., instant tea) [3]. As a result of the increasing frequency of social interactions and gatherings, particularly among younger consumers, the demand for convenient and portable tea beverages has significantly increased. This trend has significantly propelled the rapid expansion of the tea beverage market. Beverages made from green tea have gained considerable popularity among consumers, due to the tea’s refreshing, nutritious, natural, and health-enhancing properties [4,5]; these beverages also constitute a significant segment of beverage consumption in Asia, particularly in countries such as China, Japan, and India [6]. Investigations and practices have confirmed that bitterness, astringency, umami flavor, and a sweet aftertaste are the main taste attributes of green tea [7], all of which are primarily associated with polyphenols, caffeine, and amino acids [8]. Catechins, accounting for 70–80% of polyphenols and possessing antioxidant, antibacterial, and antitumor properties, are the main factors contributing to the astringency and bitterness of tea infusions [9], and also provide a distinctively important selling point of health benefits to green tea beverages [10,11,12]. Caffeine is another important source of the bitter taste of these tea infusions, while amino acids contribute to their unique umami flavor [13]. Meanwhile, volatile components, such as linalool, geraniol, and benzaldehyde, exist in extremely low abundance but play a key role in the overall quality and flavor stability of green tea beverages [14]. Thus, all the non-volatile and volatile components together constitute the unique and fascinating flavor profile of green tea beverages.

Among various types of tea beverages, green tea beverages usually face much more serious obstacles during production and storage, such as cream formation, color variation, and flavor deterioration, which significantly interfere with the quality stability and limit the shelf life of the green tea beverage. Flavor deterioration during storage is primarily manifested as a taste shift from fresh and brisk to stale and dull, as well as the loss of typical tea aroma and the emergence of unpleasant flavors [14,15]. Previous studies have indicated that significant alterations in the flavor profiles of tea beverages during long-term storage may be caused by intricate changes in their chemical constituents, including the breakdown of pigments, oxidation, and polymerization of catechins and the oxidative degradation of amino acids [16]. These chemical transformations not only directly influence the appearance of the beverage but also result in alterations in its aroma and taste attributes. The oxidation and polymerization of catechins can directly lead to beverage browning, and may also induce acidification of the matrix and release of volatiles from glycosylated precursors, thereby altering the overall flavor characteristics [17]. According to non-targeted metabolomic analysis, 37 taste-related and 3 aroma-related compounds were found to be associated with the flavor deterioration of the Da Hong Pao oolong tea beverage during storage [18]. In addition, many specific metabolites closely related to umami, astringency, and ripeness attributes were also identified from Longjing green tea beverages after accelerated thermal treatment [2]. These studies offered valuable insights into the key substances related to flavor deterioration during the storage of tea beverages. However, the limitation of the aforementioned studies is obvious, i.e., only a few tea cultivars, such as ‘Da Hong Pao’ or ‘Longjing43’ was tested, and thus the generalizability of their conclusions may be questionable. Moreover, these studies primarily documented chemical changes without exploring strategies to alleviate the undesirable flavors of tea beverages. Addressing this knowledge gap is crucial for enhancing the shelf life and market competitiveness of RTD green tea products.

Scenting is a typical process for improving the aroma of tea that is applied widely in the baked green tea market, especially [19]. Jasmine flowers are the most commonly used materials in the scenting process because of their distinctively fresh and sweet aroma. Scenting with jasmine flowers not only dramatically enhances the aroma quality of tea, but also changes the taste through the partial rehydration and re-drying of the tea that occurs during the process. In the scenting process, floral fragrance-related compounds are released from fresh jasmine flowers and then absorbed by the base tea, leading to a significant enhancement of floral compound levels in the tea [20,21]; meanwhile, the content of bitter and astringent compounds such as polyphenols in the base tea is significantly decreased through automatic oxidation and degradation, while the concentration of amino acids is increased through hydrolysis of proteins under humid-thermal conditions [22]. Inspired by these findings, we reasonably hypothesized that this scenting method has the potential to mask unpleasant off-flavors and alleviate sensory deterioration caused by COF during storage. Recently, many beverage companies have used jasmine-scented green tea to produce ready-to-drink (RTD) beverages. However, the shelf-life stability of such beverages remains largely unexplored, especially when compared with their unscented counterparts.

Previous studies have mainly focused on the individual quality deterioration of green tea products during storage, whereas few studies have addressed the cultivar differences in the storage stability of RTD tea beverages, as well as the effective regulation of cooked-off flavor (COF). In this study, sensory performance, physical and chemical properties, and volatile profiles of green tea beverages prepared from multiple tea cultivars were investigated under diverse storage conditions, and the key volatile compounds closely related to COF accumulation were screened out. Furthermore, this work preliminarily confirmed that jasmine scenting exerts a beneficial effect on alleviating storage-quality decline and restraining COF formation in tea beverages. Overall, this study reveals the variation patterns of flavor quality in stored green tea beverages and highlights the potential application of jasmine scenting for improving beverage storage stability, which can provide a feasible reference for the shelf-life maintenance of commercial ready-to-drink green tea products.

## 2. Materials and Methods

### 2.1. Chemicals

Reference compounds, including (−)-epigallocatechin gallate (EGCG), (−)-epicatechin gallate (ECG), (−)-epigallocatechin (EGC), (−)-epicatechin (EC), (−)-gallocatechin gallate (GCG), (−)-catechin gallate (CG), (−)-gallocatechin (GC), (±)-catechin (C), and caffeine (purity ≥ 99%), were purchased from Sigma-Aldrich (Shanghai, China). HPLC grade acetonitrile (ACN), methanol (MeOH), and glacial acetic acid were obtained from Merck (Darmstadt, Germany). Ninhydrin, stannous chloride, potassium dihydrogen phosphate, disodium hydrogen phosphate, potassium sodium tartrate, ferrous sulfate heptahydrate, and sodium chloride were ordered from Sinopharm Chemical Reagent Co., Ltd. (Shanghai, China). Deuterated guaiacol was purchased from Aladdin Biochemical Technology Co., Ltd. (Shanghai, China). Food-grade sodium isoascorbate and baking soda were supplied by Wuhan Nuofan Biotechnology Co., Ltd. (Wuhan, China). Pure water for preparing the beverage was obtained from Hangzhou Wahaha Group Co. (Hangzhou, China).

### 2.2. Tea Sample Preparation

**Figure 1:**
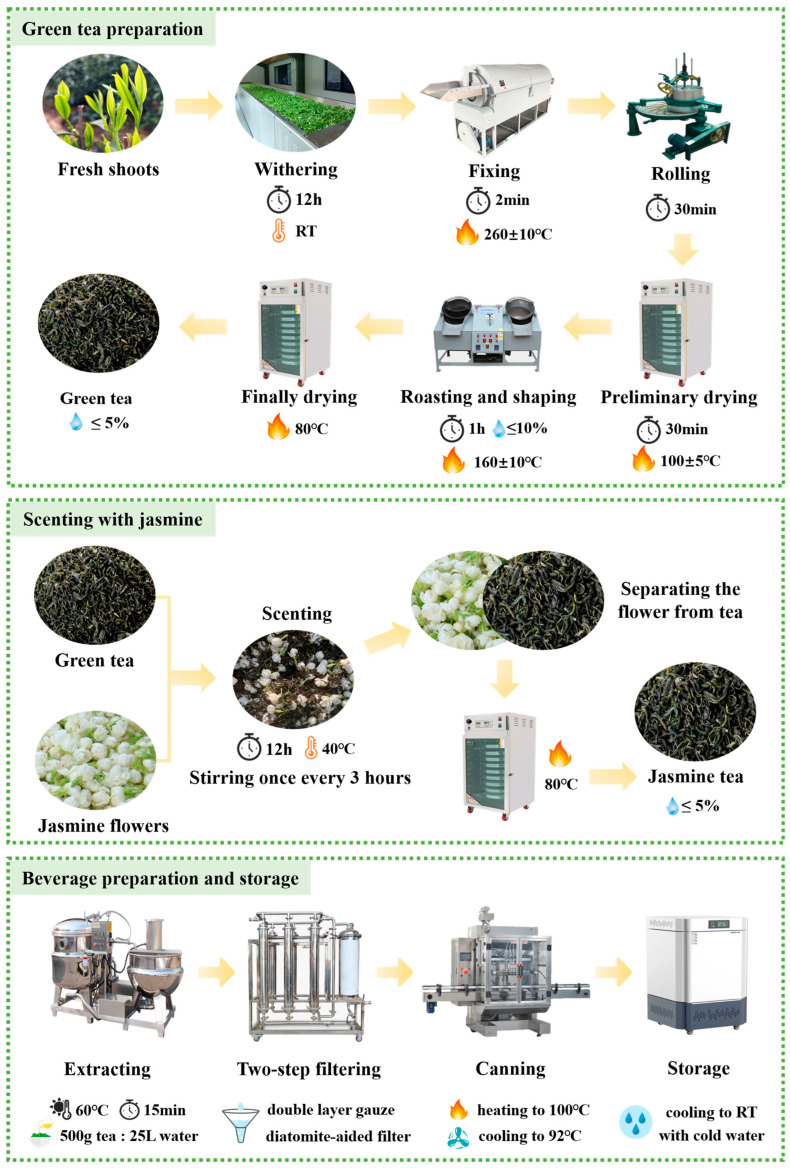
The manufacturing process of the tea and beverage.

Fresh shoots with one bud and three or four leaves were harvested in mid-May 2023 from the tea cultivars ‘Fudingdabaicha’ (FDDB), ‘Longjing43’ (LJ43), ‘Jinguanyin’ (JGY), ‘Jiukengzhong’ (JK), and ‘Zhenong117’ (ZN117) planted in the experimental tea garden of the Zhejiang University Tea Research Institute (Hangzhou, Zhejiang Province), respectively. Green tea was manufactured following standard procedures: withering at room temperature (RT) for 12 h, fixing at 260 ± 10 °C for 2 min, rolling for 30 min, drying at 100–105 °C for 30 min, roasting at 160 °C until the moisture content of the tea product in progress reaches approximately 10%, and final drying at 80 °C to below 5% moisture (Figure 1).

### 2.3. RTD Beverage Preparation and Storage

Each tea sample (500 g) was extracted with 25 L of 60 °C water containing 0.5% sodium isoascorbate and 0.1% baking soda at 60 °C for 15 min. Sodium isoascorbate and baking soda were added as permitted food additives to simulate the commercial production formula of ready-to-drink green tea beverages, and which were mainly used for antioxidant protection and pH regulation of the beverage system. The infusion was filtered through double-layer gauze and diatomite-aided filter, then diluted to a final polyphenol content of 740 mg/L. The diluted infusion was heated to 100 °C, cooled to 92 °C, and filled into sterilized PET bottles (250 mL). The filled bottle was immediately sealed with the sterilized cap, then laid down for 10 min and cooled to RT with plenty of water (Figure 1). The overall preparation procedure was modified and optimized on the basis of previous studies [16,18]. All beverage products were prepared in three independent experimental replicates to ensure the reproducibility and reliability of the subsequent analysis.

The prepared GTBs or JTBs were transferred into the dark chambers of 4 incubators for storage testing, and the temperature of the incubators was set at 4 °C, RT, 37 °C, and 55 °C, respectively. Sampling was conducted after storage for 3, 7, 14, 21, 28, 35, 42, 49, and 56 days, respectively. For the beverages stored at 55 °C, the sampling was stopped after 28 days since beverage quality had almost fully deteriorated at that time. The sampled beverages were rapidly moved to a sensory evaluation deck for temperature recovery, then sensory evaluation and physicochemical analyses were performed accordingly.

Fresh jasmine flowers were provided by Wuzhou Tea Co., Ltd. (Jinhua, China). The base tea (made from the shoots of JGY) was scented with jasmine flowers (1:1, *w*/*w*) at about 40 °C for 12 h (stirring once every 3 h). After that, the teas were sieved out from the mixture, and dried at 85 °C until to below 5% moisture.

### 2.4. Sensory Evaluation of the Beverage

The sensory quality of the beverages before and after storage was evaluated by a panel of six trained panelists, consisting of three males and three females from Zhejiang University. All members were certified by the Chinese Tea Association for their expertise in tea quality evaluation. Ethical permission was not required, and the panelists gave their consent to take part in the sensory evaluation and use their information. Sensory evaluation was conducted in strict accordance with GB/T 23776-2018 [23]. A 100-point scoring system was used for evaluation according to the weight of factors, including infusion color (25%), aroma (35%), and taste (40%), of the beverage samples. Additionally, the COF of the beverages was scored to indicate the degree of deterioration during storage using a 4-point scoring scale: 1.0 point = weak COF, 2.0 points = neutral COF, 3.0 points = strong COF, and 4.0 points = very strong COF. This scoring criteria were adapted from previous studies [2,18], with minor optimizations to better fit the context of the present study.

### 2.5. Beverage Color Difference and Turbidity Assessment

The beverage color difference indices, including the L*, a*, and b* values, were determined in transmission mode using the ColorQuest XE colorimeter (Hunter Lab, Reston, VA, USA). Total color difference (ΔE) was calculated according to the standard formula and expressed as described in [24]. When the ΔE value difference is larger than 2 units, the human eye can tell the two colors apart [25]. The turbidity of the beverage was measured by the 2100 N turbidimeter (Hach Company, Loveland, CO, USA) according to the instrument’s manual.

### 2.6. Detection of Non-Volatile Compounds

The content of total polyphenols (TPs) was determined using the ferrous tartrate colorimetric method described in GB/T 21733-2008 [26] and the content of free amino acids (FAAs) was measured using the ninhydrin colorimetric method described in GB/T 8314-2013 [27]. The absorbance values were recorded at 570 nm for TPs and at 540 nm for FAAs on an UV-1800 UV spectrophotometer (Uniko Shanghai Instrument Co., Ltd., Shanghai, China).

The content of catechins and caffeine was detected on a 20AD HPLC system (Shimadzu Corporation, Kyoto, Japan) equipped with an Agilent TC-C_18_ chromatographic column (4.6 mm × 250 mm, 5 µm). Prior to analysis, beverage samples were centrifuged at 12,000 rpm for 10 min, and the supernatant was filtered through a 0.22 µm microporous membrane. The injection volume was 10 µL, the column temperature was maintained at 35 °C, and the UV detection wavelength was set at 280 nm. The mobile phases consisted of (A) acetonitrile/acetic acid/water (30/5/965, *v*/*v*/*v*) and (B) acetonitrile/acetic acid/water (300/5/695, *v*/*v*/*v*), at a total flow rate of 1.0 mL/min. The gradient elution program was as follows: 20% B increased linearly to 80% B within the first 35 min, then B was reduced to 20% and maintained for 5 min for equilibration. Individual catechins and caffeine were identified by comparing retention times with authentic standards and quantified using external standard calibration curves. The method validation parameters, including calibration curve, correlation coefficient, limit of detection (LOD), limit of quantification (LOQ), linearity ranges, and recovery rate, are given in Table S1.

### 2.7. Analysis of Volatile Compounds

The volatile compounds were extracted from the beverages using the solid phase microextraction (SPME) technique, in which the fused silica fiber coated with 50/30 µm of divinylbenzene/carboxen/polydimethylsiloxane (Supelco, Bellefonte, PA, USA) was used. Before each extraction, the fiber head was preheated at 230 °C for 30 min in the gas chromatograph inlet to remove any remaining volatiles. A 10 mL beverage was transferred into a 20 mL headspace vial, and 100 μL of deuterated guaiacol solution (20 μg/mL) was added as the internal standard. The vial was capped immediately after the addition of 2.00 g dried sodium chloride, then moved into a thermostatic water bath (40 °C). The fiber was inserted into the vial, and the fiber tip was located 0.5 cm above the liquid level. SPME was performed at 40 °C for 50 min. After that, the fiber was pulled out from the vial and inserted into the injector of QP2010 Ultra gas chromatography–mass spectrometry (GC–MS, Shimadzu Corporation, Japan). The temperature of injector was set at 250 °C, the desorption time was 5 min. The volatiles were separated on an HP-Innowax GC column (30 m × 0.25 mm × 0.25 μm; Agilent Technologies, Palo Alto, CA, USA). Helium (purity > 99.999%) was used as carrier gas at a flow rate of 1 mL/min. The temperature program was set as follows: holding at 50 °C for 10 min, increasing to 150 °C at 3 °C/min, holding at 150 °C for 1 min, then increasing to 230 °C at 15 °C/min and holding at 230 °C for 3 min. The MS detector was set as follows: ion source temperature, 230 °C; EI ionization energy, 70 eV; scan range, 35–400 *m*/*z*. The SPME-GC-MS method was adapted from [28].

Analysis was conducted using GC Solution Workstation Software, Version 2.5 according to the manual. The volatile compounds were preliminarily identified by mass spectral matching with the NIST 11.0 database, and only compounds with a matching value above 80% were screened. Furthermore, a homologous series of n-alkanes (C7–C30) was tested under consistent chromatographic conditions to calculate the retention index (RI) of each volatile compound. The final compound qualification was comprehensively confirmed by combining experimental RI values, reference RI data from the NIST Chemistry WebBook https://webbook.nist.gov/chemistry/ (accessed on 20 March 2026), and characteristic fragment ion information. To ensure identification reliability, strict screening criteria were implemented in data processing. Peaks with ambiguous spectral information, low-quality fragment signals, and non-specific matching were manually excluded. Co-eluting components with overlapping peaks were distinguished by characteristic ion fragments and RI differences to avoid false identification. Only compounds with consistent mass spectral features, reasonable RI deviation, and stable fragment distribution were ultimately annotated. For quantitative analysis, the relative content of volatile compounds was calculated according to the following equation: *C*_x_ (μg/L) = (*A_x_*/*A_i_*) × 200, where *C_x_* (μg/L) indicates the concentration of the targeted volatile component and *A_x_* and *A_i_* represent the peak areas of the targeted volatile component and the guaiacol (internal standard), respectively. It should be noted that volatile compounds in this study were quantified via a semi-quantitative method using only guaiacol as the single internal standard. Matrix effects and compound-specific response factors were not corrected in the current analysis. Accordingly, the volatile data obtained in this work represent relative contents rather than strict absolute concentrations.

After that, the rOAV of the target volatile compound was also calculated according to the following equation: rOAV*_x_* = *C_x_*/*OT_x_*, where *OT_x_* is the odor threshold value of the target compound in water. Generally, volatile compound was considered as a key contributor to the overall aroma characteristics when its rOAV was larger than 1.

### 2.8. Data Statistics and Plotting

Data are expressed as mean ± standard deviation of triplicates. One-way ANOVA with Duncan’s multiple-range test (*p* < 0.05) was performed using SPSS 27.0 (SPSS Inc., Chicago, IL, USA). Heatmaps, line charts, radial bar charts, bar charts, stacked column charts, and correlation plots were generated with TB-tools [24] and Origin2024 (Origin Lab Corporation, Northampton, MA, USA). Venn diagrams were drawn using Jvenn https://jvenn.toulouse.inra.fr/app/index.html (accessed on 20 March 2026).

## 3. Results and Discussion

### 3.1. Change in Sensory Quality of the GTBs During Storage

**Figure 2:**
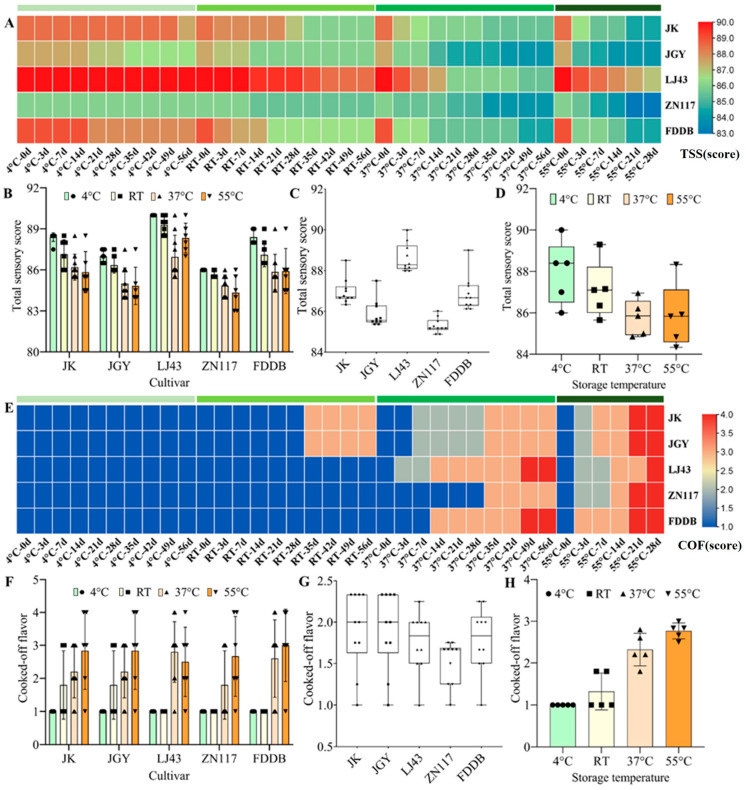
Changes in TSS and COF of the GTBs prepared from different cultivars under various storage conditions. (**A**) Heat map of the TSS. (**B**) TSS in GTBs prepared from different cultivars and stored at various temperatures. (**C**) Average of TSS in GTBs prepared from different cultivars. (**D**) Average of TSS in GTBs stored at various temperatures. (**E**) Heat map of the COF. (**F**) COF in GTBs prepared from different cultivars and stored at various temperatures. (**G**) Average of COF in GTBs prepared from different cultivars. (**H**) Average of COF in GTBs stored at various temperatures. JK. ‘Jiukengzhong’; JGY. ‘Jinguanyin’; ZN117. ‘Zhenong 117’; FDDB. ‘Fuding Dabaicha’.

To investigate the quality changes of RTD beverages during storage, we prepared GTBs from five tea cultivars and stored them at 4 °C, RT, 37 °C, and 55 °C. Initial TSS varied among cultivars: GTBs from LJ43 had the highest, followed by FDDB and JK; ZN117 had the lowest. Along with the extension in storage time, the TSS of GTBs prepared from different cultivars decreased gradually, especially when the storage was carried out at RT, 37 °C, and 55 °C (Figure 2A,B). This finding is consistent with a previous report [29]. In that study, heat treatment and long-term storage significantly influenced the sensory quality of RTD beverages. Meanwhile, the TSS of GTBs prepared from each cultivar decreased sharply with an increase in storage temperature, even for 7 days of storage. After prolonged storage at high temperatures, the differences in TSS among GTBs from different cultivars diminished. Among the five cultivars, the GTBs made from LJ43 and ZN117 exhibited relatively high-quality stability during storage, followed by the GTBs from JGY, while those from FDDB and JK were comparatively unstable (Figure 2C). Notably, LJ43 maintained a relatively high TSS with high stability, whereas ZN117 showed low TSS but high stability. Although storage temperature and time exerted dominant effects on quality deterioration, all beverage samples were treated under identical storage conditions to ensure the comparability of cultivar-related differences. Multivariate statistical analysis was not performed in the present study, and the independent and interactive effects of cultivar, storage temperature, and storage time remain to be further distinguished in future research. In addition, a significantly decreased TSS average was observed along with increased storage temperature, particularly from 4 °C to 37 °C; when the storage temperature increased from 37 °C to 55 °C, the TSS average decreased insignificantly (Figure 2D). This suggests that high temperatures make it difficult to distinguish stability differences among cultivars. Therefore, accelerated aging tests should be performed from RT to 37 °C.

In order to further measure the deterioration degree of the GTBs, the COF was scored. A higher COF score indicates greater deterioration [16]. With the extension of storage duration, the COF barely changed in all the GTBs prepared from different cultivars when storage was carried out at 4 °C, but increased slightly and differentially at RT, especially in the GTBs prepared from JK and JGY, and sharply strengthened in all GTBs from the different cultivars at 37 °C and 55 °C (Figure 2E,F). Comparatively, the GTBs of ZN117 possessed the lowest COF, followed by the GTBs of LJ43 and FDDB; those of JK and JGY had the highest COF (Figure 2G). In addition, the COF increased dramatically with the storage temperature (Figure 2H). According to the changes in COF, the deterioration of the GTBs increases with an increase in the temperature and duration of storage, which is clearly consistent with the results characterized by the TSS.

Previous studies have shown that tea beverages undergo changes in aroma compounds during storage, which leads to an imbalance in overall aroma and the development of off-flavors, with COF being the most prevalent [30]. In the process of taste perception, olfaction and gustation interact to influence taste perception through cross-sensory interactions [31]. Consequently, this interaction mechanism further diminished the overall flavor quality of tea beverages by generating COF during storage. The above results indicated that the generation of COF during the storage of GTBs would further reduce the overall flavor quality of the tea beverages.

### 3.2. Changes in Color and Turbidity of the GTBs During Storage

**Figure 3:**
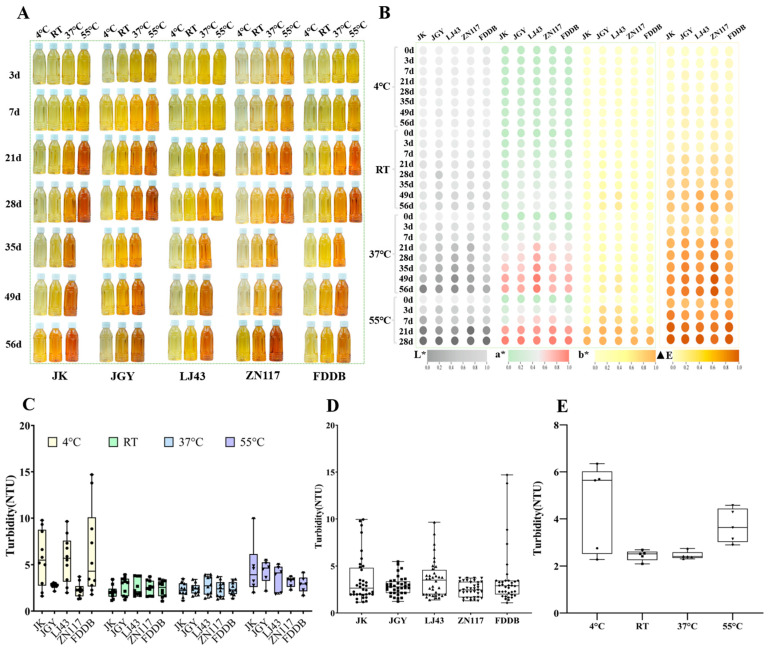
Color and turbidity changes of GTBs under different storage conditions. (**A**) Appearance of GTBs; (**B**) The heatmap of L*, a*, b*, and ΔE values of GTBs. The visualization was performed using TBtools, Version 2.420. after the data were normalized. (**C**) Turbidity in the GTBs prepared from different cultivars and stored at various temperatures; (**D**) Average turbidity in GTBs prepared from different cultivars; (**E**) Average turbidity in GTBs stored at different temperatures.

The appearance and color difference (L*, a*, b*, and ΔE) of the GTBs under different storage conditions were recorded at each sampling point. The GTBs freshly prepared from different cultivars were all yellowish-green in color, although there were some subtle differences among the various cultivars. Along with an increase in storage temperature and extension of the storage duration, browning of the GTBs gradually increased. Browning took place very slowly at temperatures not higher than RT, but occurred very quickly at 37 °C and 55 °C. In general, the GTBs prepared from LJ43 exhibited the slowest browning during storage (Figure 3A). The L* value continuously declined with an increase in storage time, while the a*, b*, and ΔE values steadily rose; within the same storage duration, the higher the temperature, the greater the variations in the L*, a*, b*, and ΔE values of the beverages (Figure 3B). These results are consistent with previous reports [16,32]. The observed color deterioration can be explained by several concurrent reactions. The oxidation and degradation of phenolic compounds, along with the formation of colored Maillard reaction products, were the primary drivers of color changes in GTBs during storage [33,34]. In addition, non-enzymatic oligomerization of epicatechin and catechin at elevated temperatures produced dehydrodicatechin A, which induced browning in model systems [35]. Recent evidence also showed that baicalin and ascorbic acid helped maintain brightness, whereas flavonoids, flavonoid glycosides, and oxidized catechin derivatives contributed to darkening and hue shifts during storage [36]. Consistent with these mechanisms, our data (Section 3.3) showed a marked decrease in catechins, especially EGCG, supporting the role of phenolic oxidation in the observed browning.

Turbidity is widely regarded as a crucial parameter for the sensory quality of tea beverages. In our research, all GTBs consistently maintained relative clarity and transparency without remarkable cream or precipitation issues. Measurement showed that the turbidity varied with cultivar and storage temperature. In detail, the turbidity in the GTBs prepared from JGY and ZN117 was lower than that from FDDB, JK, and LJ43, indicating that the clarity of tea beverages varied among different cultivars (Figure 3C,D). This phenomenon could be attributed to the differences in the proportions of polyphenols and proteins contributing to the formation of tea-cream among the different cultivars of GTBs, which directly influenced the formation rate and quantity of the aggregates [37,38]. Meanwhile, relatively higher turbidity was observed in the GTBs stored at 4 °C, followed by GTBs stored at 55 °C, and relatively lower turbidity was observed in the GTBs stored at RT and 37 °C (Figure 3E). The high turbidity at 4 °C is likely due to the hydrogen bond-driven assembly of polyphenols and proteins, while that at 55 °C arises from oxidative polymerization of catechins producing insoluble particles. Previous studies have demonstrated that tea-cream formation primarily results from non-covalent interactions (e.g., hydrogen bond and hydrophobic effects) among its constituents. Since hydrogen bond strength decreases with increasing temperature, lower temperatures are more conducive to the formation of tea cream [39]. During storage of GTBs, the increasing oxidation degree of catechins significantly enhanced their capacity to participate in tea-cream formation. Under 55 °C storage conditions, the formation of catechin oxidation products directly contributed to beverage turbidity development. As shown in Figure 3E, the non-monotonic turbidity response (highest at 4 °C, moderate at 55 °C, lowest at RT/37 °C) supports the proposed dual-mechanism interpretation. It is worth mentioning that the highest turbidity in all GTBs was around 15 NTU, which was lower than the level perceptible to the naked eye (50 NTU). Therefore, these GTBs were relatively stable and were acceptable from the perspective of turbidity.

### 3.3. Changes in Taste-Related Components of the GTBs During Storage

**Figure 4:**
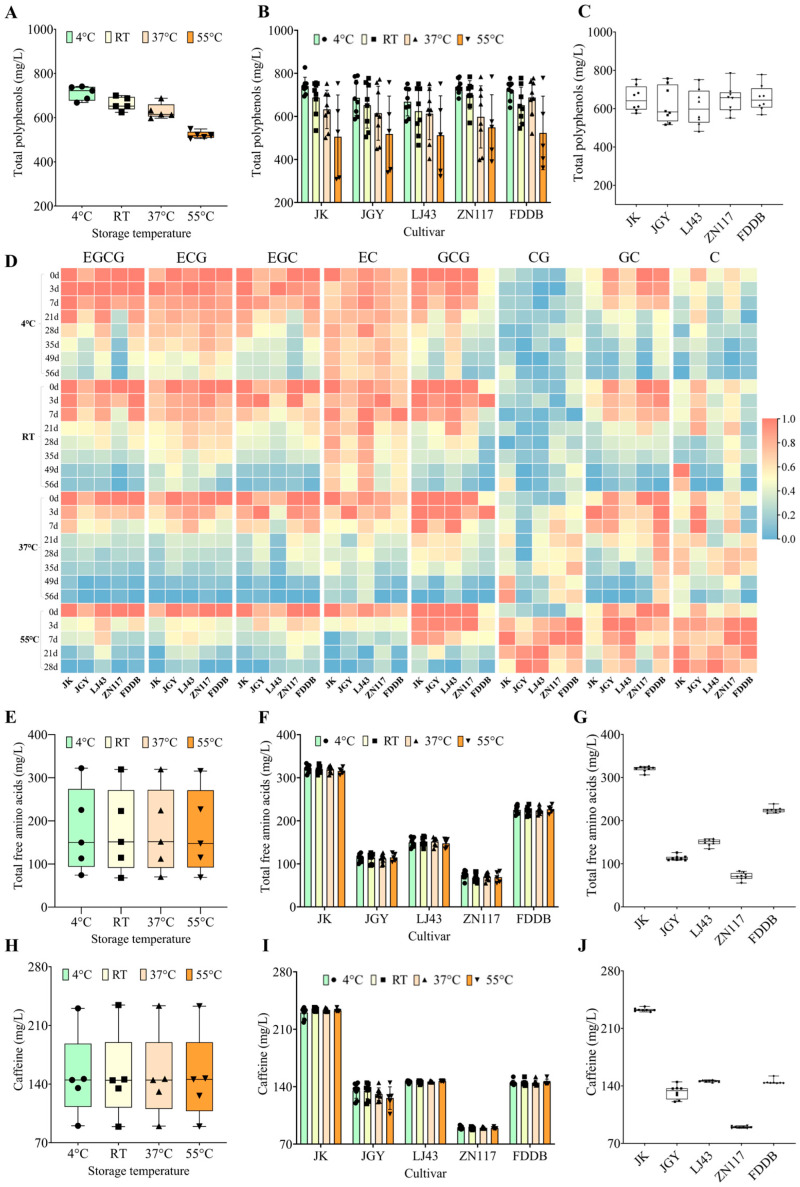
Dynamic changes in taste-related components of the GTBs under different storage conditions. (**A**) Average content of TPs in GTBs stored at various temperatures. (**B**) Content of TPs in GTBs prepared from different cultivars and stored at various temperatures. (**C**) Average content of TPs in GTBs prepared from different cultivars. (**D**) Change in content of catechins during storage. (**E**) Average content of FAAs in GTBs stored at various temperatures. (**F**) Content of FAAs in GTBs prepared from different cultivars and stored at various temperatures. (**G**) Average content of FAAs in GTBs prepared from different cultivars. (**H**) Average content of caffeine in GTBs stored at various temperatures. (**I**) Content of caffeine in GTBs prepared from different cultivars and stored at various temperatures. (**J**) Average content of caffeine in GTBs prepared from different cultivars.

TPs and catechins were considered as crucial flavor compounds [11,40,41] determining the taste of the GTBs during processing and storage [17]. The results show that the content of TPs decreased with an increase in storage time and temperature (Figure 4A–C), which is consistent with the previous report [42]. The level of TPs was also influenced by cultivars, especially at relatively low storage temperatures. During storage at 4 °C, the lowest decline rate (7.87%) of TPs was observed in the GTBs prepared from JK, while the highest (23.21%) was witnessed in the GTBs from LJ43. The GTBs of ZN117 exhibited the least reduction in TPs (27.34%) under the RT storage condition, while the GTBs of JK and FDDB displayed relatively minor decreases in TPs under the 37 °C storage condition. The decrease in TPs may be attributed to the oxidation of catechins and precipitation with proteins [14], but we did not identify specific reaction products. Future studies using LC-MS/MS or high-resolution mass spectrometry are needed to characterize the actual reaction intermediates and products.

HPLC analysis confirmed that the content of most catechin monomers exhibited a gradual decline with prolonged storage duration. Notably, EGCG showed the most significant decrease among these components. Meanwhile, the level of EGCG decreased sharply with an increase in storage temperature (Figure 4D). This indicates that the decrease in TPs mainly resulted from the reduction of catechins during storage. The significant color change combined with only a minor turbidity increase suggests that catechin loss was mainly due to oxidative or thermal degradation rather than precipitation. This interpretation is supported by [17], who found that heat treatment of tea beverages led to catechin degradation rather than cream formation. It is worth pointing out that the impact of heat treatment or storage on monomeric catechin content in the GTBs is not solely determined by temperature alone, but also by a synergistic effect of temperature and duration [2].

FAAs have been considered an important indicator for assessing tea quality contributing to the freshness and umami of tea beverages [43]. Analysis showed that no significant change in the FAA content was observed under different storage conditions in the GTBs initially prepared from shoots of the same cultivar (Figure 4E,F), suggesting that the Maillard reaction might not be a major contributor to beverage browning during storage. Notably, considerable variations were observed in the FAAs levels among the GTBs prepared from shoots of different cultivars. In particular, the GTBs of ZN117 and JGY possessed relatively lower FAA levels, while those of JK and FDDB had higher FAA levels (Figure 4G). Since the initial TPs concentration was standardized across all beverages, lower FAA results in a higher TP/FAA ratio, a critical index determining the taste quality of green tea. A lower TP/FAA ratio is associated with better freshness and umami (suitable for high-quality green tea), whereas a higher ratio corresponds to increased bitterness and astringency [44]. Therefore, the lower sensory scores of ZN117 and JGY GTBs can be attributed to their higher TP/FAA ratio. This suggests that tea cultivars rich in free amino acids are more suitable for producing GTBs with desirable taste.

Similar to FAAs, caffeine level remained relatively stable in the GTBs prepared from the shoots of the same cultivar under specific storage conditions, but varied significantly in the GTBs prepared from different cultivars (Figure 4H–J). The lowest caffeine content was observed in the GTBs from ZN117, while the highest was found in those from JK. Caffeine was a crucial flavor contributing to the bitterness of the beverage [45]. Many previous studies also showed caffeine maintained its stability during the storage of tea beverages [14,17]. Consequently, caffeine level is unlikely to be related to flavor or color deterioration in our system.

### 3.4. Change in Aroma-Related Components of the GTBs During Storage

**Figure 5:**
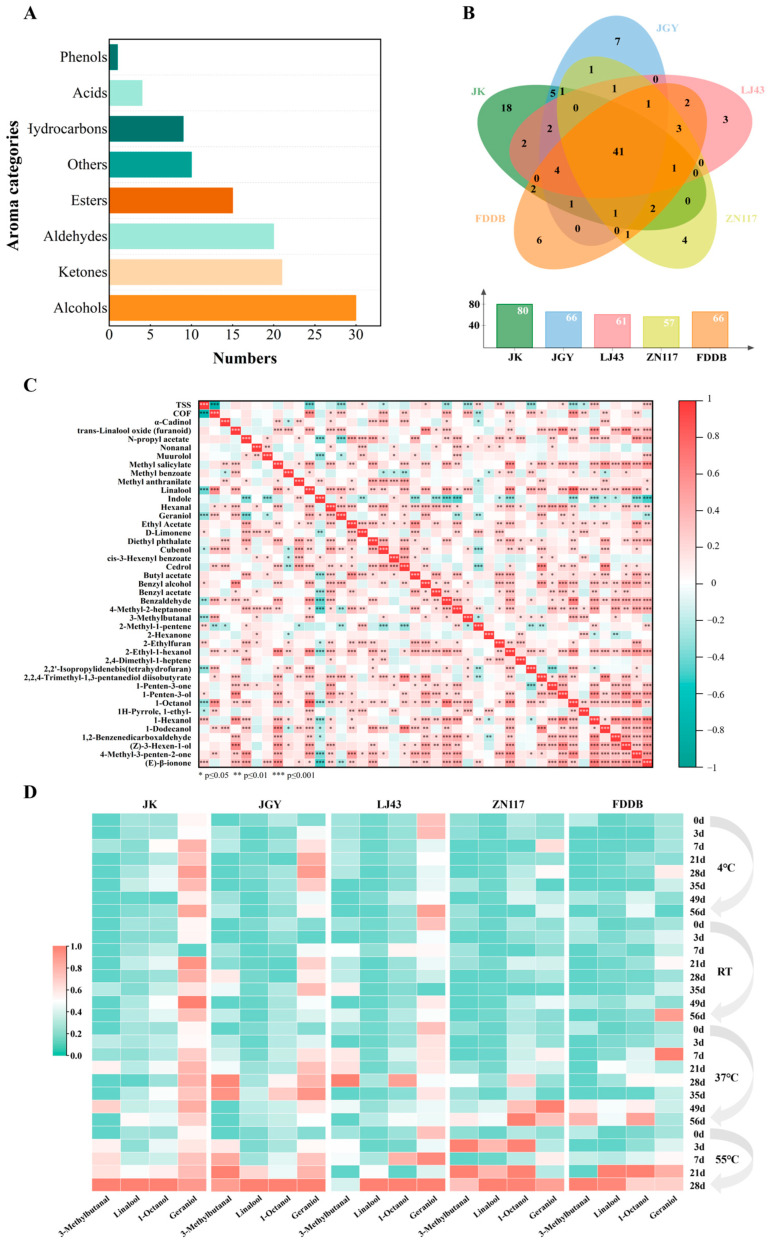
The key volatiles related to COF in GTBs according to rOAV and Spearman correlation analysis. (**A**) The aroma categories of all volatile components in GTBs under different storage conditions. (**B**) Volatile number in different GTBs. (**C**) Spearman correlation analysis among the dynamic change in level of the shared volatiles, TSS and COF. (**D**) Dynamic changes of the level of key volatiles related to COF during storage.

According to the SPME-GC-MS analysis, a variety of aroma components were observed in the GTBs, and these significantly changed during storage. A total of 110 volatile compounds were identified, including 30 alcohols, 21 ketones, 20 aldehydes, 15 esters, 9 hydrocarbons, 4 acids, 1 phenol, and 10 other compounds (Figure 5A). From the perspective of abundance, alcohols, esters, and aldehydes were the main components (Figure S1), which was consistent with previous reports [46,47]. Among the GTBs prepared from different cultivars, JK and JGY showed the highest total volatiles levels, followed by FDDB, while LJ43 and ZN117 had the lowest (Figure S1). Although the concentrations and proportions of various volatiles changed during storage, these changes did not show strong correlations with storage time or temperature. This indicates that the genetic and biochemical differences among cultivars played a dominant role in determining the aroma composition of the GTBs.

COF is a prevalent off-flavor negatively correlated with TSS and commonly found in hot-processed foods [48]. To elucidate the key metabolites responsible for COF formation during storage, a total of 41 common volatiles were identified from all the GTBs from five cultivars (Figure 5B) and used to conduct the correlation analysis with the COF and TSS of beverages (Figure 5C). Spearman correlation analysis identified 15 volatiles positively correlated with COF (*p* < 0.05) and 15 volatiles negatively correlated with TSS (*p* < 0.05). Among these, nine volatiles were both positively correlated with COF and negatively correlated with TSS, including linalool, geraniol, cubenol, benzaldehyde, 3-methylbutanal, 2-methy-1-pentene, 2,2′-isopropylidenebis(tetrahydrofuran), 1-octanol, and 1-ethyl-1H-pyrrole. These nine compounds are likely key contributors to flavor deterioration during the storage of beverages.

The contribution of volatile substances to the overall aroma of tea beverages is determined by the ratio of the relative content to the corresponding odor threshold (rOAV), with volatiles exhibiting rOAV > 1 generally considered as key aroma contributors [49,50]. Among the nine volatiles, the contribution of the cubenol, 2-methyl-1-pentene, and 2,2′isopropylidenebis (tetrahy-drofuran) could not be determined because of unavailable odor thresholds, while the contribution of benzaldehyde and 1-ethyl-1H-pyrrole might be ignored because of their rOAVs < 1. In contrast, 3-methylbutanal, linalool, 1-octanol, and geraniol significantly contributed to the COF since their rOAVs were greater than 1 during the storage period (Table S2), especially at the later period of storage. A heat map was plotted in order to visualize the variation in the key volatiles for COF along with storage processes (Figure 5D). Specifically, at 37 °C and 55 °C, the concentration of these volatiles increased significantly with an extension in storage time. The accelerated accumulation at 55 °C (Figure 5D) compared with 37 °C suggests that temperature strongly promotes either the release of these volatiles from precursors or their thermal generation pathways, consistent with the higher COF scores at 55 °C (Figure 2H). Therefore, the temperature-dependent accumulation of these four volatiles, which parallels the rise in COF scores at elevated temperatures, further supports their role as key contributors to COF.

The accumulation of these four volatiles during storage arises from distinct chemical pathways. The increase in linalool and geraniol after high-temperature storage can be explained by their release from non-volatile precursors. Previous studies demonstrated that glycosidically bound volatiles or linalyl/geranyl pyrophosphates underwent thermal hydrolysis, liberating free linalool and geraniol [51]. Similar observations have been reported in different types of tea infusions during heat treatment [18,52]. A more detailed mechanistic linkage between catechin degradation and the accumulation of these terpene alcohols was provided by Dou et al. [14]. According to their study, the significant increase in linalool during storage is primarily caused by the oxidative degradation of catechins, which promotes extensive hydrolysis of glycosidically bound terpenoids and aromatic alcohols. Furthermore, linalool can undergo isomerization to nerol, which subsequently converts to geraniol, thereby accounting for the concurrent increase in geraniol content. In the present study, we observed a pronounced decline in total polyphenols and catechins (Figure 4A–D) under elevated storage temperatures (37 °C and 55 °C), accompanied by a marked accumulation of linalool, geraniol, 1-octanol, and 3-methylbutanal (Figure 5D). The parallel trends between catechin degradation and COF-related volatile accumulation, together with the isomerization pathway from linalool to geraniol, support the interpretation that catechin oxidation serves as a key chemical driver linking non-volatile deterioration to cooked-off flavor formation in green tea beverages during storage. The formation pathways of 3-methylbutanal and 1-octanol are preliminarily speculated based on the previous literature. 3-Methylbutanal is widely recognized as a typical Strecker aldehyde mainly generated from leucine degradation during thermal processing and storage [29,53]. 1-Octanol is generally considered to be derived from lipid oxidation or the hydrolysis of glycosidic precursors [54], though it may also be released from glycosidic precursors under thermal stress, since another C8 alcohol, 1-octen-3-ol, has been considered an off-flavor compound in soybean products and confirmed to be formed from hydrolysis of its β-primeveroside [55,56]. However, the corresponding precursor indicators (e.g., free amino acid consumption or lipid oxidation products) were not determined in the present study. Therefore, the above formation mechanisms remain to be further verified by more targeted detection in future research. Taken together, these results indicate that elevated temperatures combined with prolonged storage trigger a series of chemical reactions. These include glycosidic hydrolysis, Strecker degradation, and fatty acid derived pathways. Such reactions lead to the accumulation of 3-methylbutanal, 1-octanol, geraniol, and linalool. The high concentrations of these four volatiles may be the main contributors to COF in GTBs.

Under normal conditions, linalool, geraniol, 3-methylbutanal, and 1-octanol each contribute pleasant odor attributes. Linalool and geraniol typically provide floral and citrus-like notes [57,58]; 3-methylbutanal imparts a malty, roasted aroma [53]; and 1-octanol presents a green, grassy, and sweet floral scent [54]. During storage, however, the substantial accumulation of these volatiles alters the volatile composition and proportions, resulting in an overall aroma profile that deviates from that of the fresh beverage. This sensory imbalance, rather than the presence of any inherently offensive compound, is perceived by consumers as a COF or a stale flavor. As previously reported by Kinugasa and Takeo [59], the cooked-off flavor results from the loss of fresh, green volatiles and the simultaneous release of linalool and geraniol from their glycosidic precursors. This mechanism was further confirmed by a recent sensomics study on Biluochun green tea beverages, which identified linalool and geraniol as key contributors to COF [30]. Therefore, the identification of these four volatiles as COF contributors reflects their role in the distorted aroma balance under storage conditions, not an intrinsic change in their odor quality. Their concurrent accumulation synergistically alters the original volatile profile and culminates in the characteristic COF.

### 3.5. Influence of Scenting on Physical and Chemical Properties Changes of the GTBs

Scenting can significantly improve the overall quality of green tea through enhancing the aroma profile and richness of tea as well as reducing its bitterness [60,61]. Jasmine tea was prepared after scenting the jasmine flower with the JGY green tea and processed into beverages (JTBs). Subsequently, both the JTBs and control GTBs prepared from JGY green tea were subjected to the storage treatments described above (Figure S2A). Under identical conditions, JTBs had significantly higher TSS than GTBs throughout storage, although TSS declined over time in both. The increase in COF with temperature and time was much lower in JTBs than in GTBs (Figure S2B). In comparison with GTBs, JTBs exhibited relatively small changes in brightness and total color difference during storage, though decreased L* value but increased a*, b*, and ΔE values were observed (Figure S2C). These observations suggest that beverages prepared from jasmine-scented green tea have better sensory and color stability than unscented ones. During storage, change trends of taste-related compounds in JTB was similar to those in GTBs. The content of TPs showed an overall downward trend, while the levels of caffeine and FAAs exhibited fluctuations within a small range (Figure S3). It is worth noting that, with increased temperature and prolonged time, the decrease in TP content in JTBs was significantly smaller than that in GTBs. The underlying chemical basis for this difference remains unclear based on the present data. Future studies should perform comparative metabolomic profiling of tea infusions before and after jasmine scenting to identify jasmine-derived compounds (e.g., specific phenolic acids or other stabilizers) that may account for the protective effect. The lack of such metabolomic analysis is a limitation of this study, and further investigation is needed to elucidate the precise mechanism.

**Figure 6:**
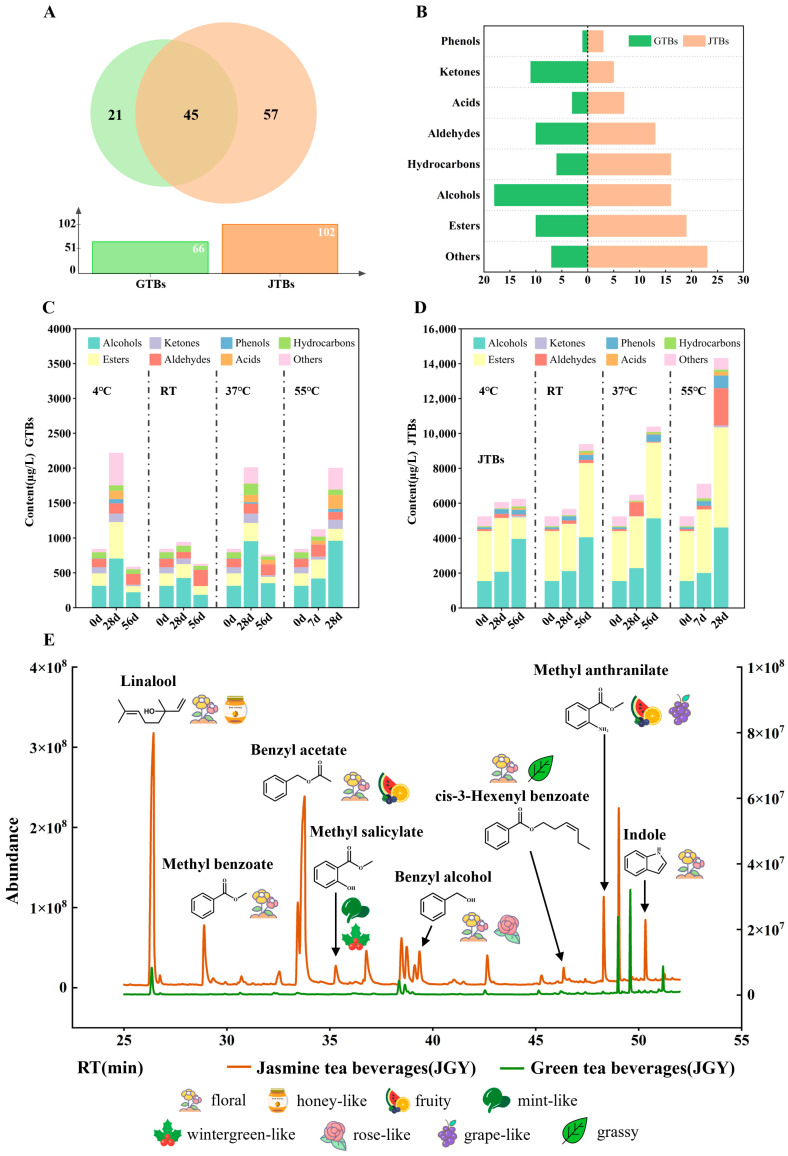
Comparative analysis of volatiles in GTBs (JGY) and JTBs (JGY). (**A**) Comparison of the volatile number between GTBs (JGY) and JTBs (JGY). (**B**) Comparison of the types of volatiles between GTBs (JGY) and JTBs (JGY). (**C**) Typical total ion current spectrum obtained from JTBs and GTBs (JGY). (**D**) Content variations of the different volatiles in GTBs (JGY) stored at early, middle, and late stages. (**E**) Content variations of the different volatiles in JTBs (JGY) stored at early, middle, and late stages.

SPME-GC-MS analysis showed that JTBs and GTBs from JGY contained 57 and 21 unique volatiles, respectively, alongside 45 shared compounds (Figure 6A), implying that jasmine scenting significantly endowed JTBs with a wider variety of volatiles, especially a higher frequency of esters, aldehydes, hydrocarbons, acids, and other compounds compared with GTBs (Figure 6B). During storage, GTBs showed an initial increase followed by a decrease at 4–37 °C (Figure 6C). In contrast, the total volatile content in JTBs increased markedly with longer time and higher temperature (Figure 6D). This increase was largely attributable to the accumulation of alcohols and esters, especially at temperatures above RT. Comparison of the GC-MS total ion current chromatograms between JTBs and unscented GTBs revealed that JTBs contained much higher levels of several specific volatiles (Figure 6E). The major volatiles in JTBs included linalool, benzyl acetate, methyl benzoate, methyl anthranilate, indole, methyl salicylate, (Z)-3-hexen-1-ol, benzyl alcohol, and geraniol. These compounds are characteristic aroma components of jasmine-scented green tea, as demonstrated in previous studies [20,62]. Among these volatiles, linalool, methyl benzoate, benzyl acetate, benzyl alcohol, cis-3-hexenol benzoate, and indole contribute floral notes; methyl salicylate has a holly-like odor; and methyl anthranilate provides fruity and grape-like aromas [58,63,64]. From a biosynthetic perspective, these volatile compounds originate from different precursors, with amino acids and glycosidic conjugates playing dominant roles [65]. It is therefore plausible that the jasmine-scenting process introduced additional precursors, including free amino acids and glycosides, into the tea leaves. These precursors could subsequently release the corresponding volatiles under thermal stress during storage. However, direct experimental evidence, such as quantification of glycosidic precursors and free amino acid profiling before and after storage, is lacking. This hypothesis therefore requires further verification.

In addition to overall aroma profiling, we specifically monitored the changes of the four identified COF-related volatiles (linalool, 1-octanol, 3-methylbutanal, and geraniol) during storage of the JTBs (Figure S4). The basal contents of linalool and 1-octanol were higher in JTBs than in GTBs, but their change trends were similar, indicating that jasmine scenting increased initial levels but did not alter storage behavior. Importantly, although 3-methylbutanal in JTBs exhibited a similar basal content and change trend to that in GTBs of JGY, it increased on a much smaller scale along with temperature increase and duration extensions, suggesting that the increase in 3-methylbutanal levels was significantly suppressed during storage of JTBs. In addition, scenting did not significantly affect the basal level or storage behavior of geraniol. Based on these observations, the lower COF in JTBs can be explained by two complementary mechanisms. First, the suppression of 3-methylbutanal formation directly reduces the concentration of this known off-flavor compound. Second, the abundant floral and fruity volatiles introduced by jasmine scenting (such as linalool, benzyl acetate, and indole) may exert a masking effect, reducing the perceptual impact of any remaining COF-related odors. Together, these two factors contribute to the improved flavor stability of JTBs.

These findings demonstrate that jasmine scenting is an effective strategy for extending the shelf life of green tea beverages. It enhances the volatile profile and reduces the formation of the key off-flavor compound 3-methylbutanal. In addition, JTBs exhibited a smaller decrease in total polyphenols during storage compared with GTBs. Although the mechanism remains unclear, one plausible explanation is that the abundant aromatic compounds from jasmine flowers may compete with polyphenols for oxidation or form complexes that stabilize polyphenols. This hypothesis requires experimental verification.

## 4. Conclusions

The changes in sensory, physical, and chemical properties, along with the formation of COF and its alleviation, were systematically investigated in infusions prepared from five green teas and one jasmine tea stored under different conditions. Decreased TSS, increased COF, and increased ΔE were observed with increasing temperature and time in all GTBs, especially at higher temperatures and longer storage times. The GTBs prepared from shoots of LJ43 and ZN117 exhibited higher quality stability than those prepared from other cultivars. Beverage accelerated aging tests should be performed at temperatures ranging from RT to 37 °C, since it is easier to distinguish the GTB difference among various cultivars. The marked decrease observed in TPs and catechins, in contrast to the stability of caffeine and FAAs, indicates that the oxidative degradation of phenolic compounds is closely associated with quality deterioration of GTBs. In addition, jasmine-scented beverages showed a smaller decrease in TPs during storage compared with their unscented counterparts, although the mechanism underlying this protective effect remains unclear. Genetic and biochemical differences among cultivars played a dominant role in determining the aroma quality and variation behaviors of volatiles during storage. COF development is due to the accumulation of linalool, geraniol, 1-octanol, and 3-methylbutanal during storage. Lower COF in JTBs is likely associated with the reduction of 3-methylbutanal, as well as changes in the proportions of the volatiles. In brief, this study systematically revealed the quality deterioration mechanism of GTBs from the perspective of physical and chemical properties and volatile flavor metabolism, and further clarified the key volatile compounds driving COF formation. More importantly, the present work confirmed the positive effect of jasmine scenting on retarding flavor deterioration, which provides a novel insight and practical technical reference for the quality maintenance and shelf-life extension of ready-to-drink green tea beverages.

## Data Availability

Dataset available on request from the authors: the raw data supporting the conclusions of this article will be made available by the authors on request.

## Supplementary Materials

The following supporting information can be downloaded at: https://www.mdpi.com/article/10.3390/foods15101656/s1, Table S1. Method validation for detection of catechins and caffeine through HPLC. Table S2. Relative odor activity values of the key volatiles related to COF in GTBs. Figure S1. Changes in level of volatiles in GTBs during storage. Figure S2. Changes in appearance, sensory quality, color difference of JTBs and GTBs (JGY) during storage. Figure S3. Dynamic changes in non-volatile components in JTBs and GTBs (JGY) during storage. Figure S4. Change in the key vola-tiles related to COF in JTBs and GTBs (JGY) during storage.
