# Supplementary material for: Physical and Chemical Property Changes, Cooked-Off Flavor Formation, and Its Alleviation During Storage of Green Tea Beverages
Source: Foods. 2026 May 9;15(10):1656. doi: 10.3390/foods15101656 (PMC13205239; doi:10.3390/foods15101656)
Supplement: Supplementary file 1 [file foods-15-01656-s001.zip › foods-4282782-supplementary.pdf]

Supplementary files

Table S1. Method validation for detection of catechins and caffeine through HPLC.

Equation	Correlation coefficient	LOD(μg/ml)	LOQ(μg/ml)	The linearity ranges(μg/mL)	Recovery(%)
$Y_{(GC)}$ =1.2758E-05*$X_{(GC)}$-6.9402E-04	r=0.9941	0.225	0.751	2.25-400	92-108
$Y_{(EGC)}$=9.5849E-06*$X_{(EGC)}$-5.6224E-05	r=0.9999	0.176	0.585	1.76-400	95-104
$Y_{(C)}$=3.1020E-06*$X_{(C)}$+1.8820E-04	r=1.0000	0.072	0.240	0.72-400	93-105
$Y_{(EC)}$=3.2896E-06*$X_{(EC)}$-4.0698E-04	r=0.9977	0.069	0.231	0.69-400	98-102
$Y_{(EGCG)}$=1.7962E-06*$X_{(EGCG)}$+7.9884E-05	r=0.9999	0.042	0.140	0.42-1600	99-103
$Y_{(GCG)}$=1.4987E-06*$X_{(GCG)}$+1.1568E-04	r=0.9999	0.035	0.116	0.35-400	99-104
Y(ECG)=1.3760E-06*X(ECG)+7.9884E-05	r=0.9999	0.036	0.121	0.36-1600	98-103
$Y_{(CG)}$=1.2230E-06*$X_{(CG)}$+7.0616E-05	r=0.9999	0.031	0.104	0.31-400	99-104
$Y_{(Caffeine)}$= 3.5215E-05*$X_{(Caffeine)}$	r=0.9999	0.019	0.057	0.19-1200	98-102

Note: Y. Concentration of the target compound (μg/ml); X. Peak area of the target compound at 280nm; LOD. Limit of detection; LOQ. Limit of quantification.

Table S2. Relative odor activity values of the key volatiles related to COF in GTBs.

Volatiles	Odor threshold (μg/L)	Odor descriptions	Temperature	rOAV in beverage of JK			rOAV in beverage of JGY			rOAV in beverage of LJ43			rOAV in beverage of ZN117			rOAV in beverage of FDDB		
				Min	Max	Average	Min	Max	Average	Min	Max	Average	Min	Max	Average	Min	Max	Average
3-Methylbutanal	0.5[a]	Malty	4°C	0.00	65.07	13.01±29.10	0.00	34.26	23.69±14.18	0.00	21.54	15.29±7.95	14.96	26.83	19.45±5.28	44.59	51.34	47.97±4.77
			RT	0.00	80.89	16.18±36.18	0.00	76.42	30.05±29.02	0.00	46.57	25.93±15.91	16.43	22.72	20.13±3.29	41.31	51.34	45.87±5.08
			37°C	0.00	228.74	110.62±94.26	0.00	126.94	54.72±52.06	0.00	84.19	34.09±30.95	16.43	73.84	37.44±24.49	51.34	155.94	107.89±52.81
			55°C	0.00	334.07	187.76±120.04	0.00	130.59	86.76±52.23	15.46	42.05	31.58±11.98	16.43	127.20	91.38±52.26	51.34	196.37	101.94±81.85
Linalool	0.22[b]	Floral, sweet,	4°C	145.38	253.03	194.70±33.40	68.70	212.37	128.82±56.81	91.67	154.50	117.39±21.64	70.87	108.20	93.70±12.37	66.89	117.62	82.16±17.20
		grape-like,	RT	173.04	251.69	206.87±30.01	82.20	205.26	123.94±46.39	94.55	190.16	121.65±29.96	64.63	133.15	91.02±23.34	73.00	121.05	93.34±16.74
		woody	37°C	136.72	343.65	229.19±59.36	104.95	689.44	381.19±203.55	118.17	285.40	170.48±66.51	91.09	611.00	241.01±209.19	70.53	264.40	157.70±85.49
			55°C	187.17	536.81	303.58±141.29	205.26	1989.94	828.10±800.21	110.78	853.81	360.68±309.25	91.09	1254.00	688.84±549.67	69.00	471.35	247.76±202.27
Cubenol	n.f.	n.f.	-	-	-	-	-	-	-	-	-	-	-	-	-	-	-	-
Benzaldehyde	350[b]	Almond-like,	4°C	0.00	0.16	0.11±0.05	0.07	0.86	0.26±0.26	0.01	0.17	0.08±0.06	0.02	0.08	0.04±0.02	0.10	0.24	0.17±0.05
		fruity,	RT	0.00	0.17	0.11±0.05	0.02	0.25	0.09±0.07	0.02	0.17	0.06±0.05	0.02	0.06	0.03±0.01	0.04	0.23	0.15±0.06
		cherry-like	37°C	0.06	0.14	0.11±0.03	0.00	0.65	0.17±0.20	0.02	0.17	0.06±0.05	0.02	0.08	0.06±0.02	0.04	0.26	0.16±0.06
			55°C	0.11	0.15	0.12±0.02	0.07	0.43	0.19±0.15	0.03	0.17	0.10±0.06	0.02	0.07	0.06±0.02	0.06	0.17	0.13±0.04
2-Methyl-1-pentene	n.f.	n.f.	-	-	-	-	-	-	-	-	-	-	-	-	-	-	-	-
2,2'-Isopropylidenebis (tetrahydrofuran)	n.f.	n.f.	-	-	-	-	-	-	-	-	-	-	-	-	-	-	-	-
1-Octanol	0.02[b]	Green, citrus,	4°C	229.07	649.48	447.34±141.49	123.84	387.27	261.50±84.43	337.28	609.53	415.97±82.38	135.02	374.10	245.18±83.17	240.62	610.20	364.52±141.95
		fatty,	RT	330.19	631.01	469.43±110.26	92.83	387.27	280.22±113.30	323.35	859.94	462.19±168.81	117.27	361.91	218.56±91.25	271.59	508.86	351.73±81.59
		coconut-like	37°C	363.56	625.46	496.34±104.80	365.69	1105.47	562.96±266.38	348.55	1261.15	619.85±296.88	229.11	974.00	485.42±267.70	258.86	980.19	555.56±247.02
			55°C	366.65	1052.49	637.94±260.43	316.02	1552.20	710.74±494.52	218.94	1407.51	698.90±550.12	229.11	969.35	674.16±399.44	296.85	1093.48	627.42±332.61
Geraniol	7.5[b]	Rose-like,	4°C	3.16	5.48	4.38±0.92	3.33	32.39	20.69±9.17	2.57	5.55	3.68±1.18	1.49	6.75	3.81±1.82	0.53	4.01	1.83±1.04
		sweet,	RT	3.31	6.21	4.59±1.16	3.33	27.30	17.58±7.92	2.08	4.74	3.25±0.82	1.65	5.06	3.12±1.10	1.31	5.92	2.76±1.50
		honey-like	37°C	3.22	5.12	4.34±0.73	3.33	33.33	21.03±9.46	2.78	4.74	3.58±0.66	1.78	9.81	4.72±2.87	1.66	6.60	2.83±1.64
			55°C	3.31	5.62	4.39±0.91	3.33	36.24	22.82±12.50	2.38	6.37	4.48±1.85	2.60	8.82	4.96±2.53	1.66	5.50	3.68±1.53
1-Ethyl-1H-pyrrole	10000[b]	Burnt, roasted	4°C	0.00	0.00	0.00	0.00	0.00	0.00	0.00	0.00	0.00	0.00	0.00	0.00	0.00	0.00	0.00
			RT	0.00	0.00	0.00	0.00	0.00	0.00	0.00	0.00	0.00	0.00	0.00	0.00	0.00	0.00	0.00
			37°C	0.00	0.00	0.00	0.00	0.00	0.00	0.00	0.00	0.00	0.00	0.00	0.00	0.00	0.00	0.00
			55°C	0.00	0.00	0.00	0.00	0.00	0.00	0.00	0.00	0.00	0.00	0.00	0.00	0.00	0.00	0.00

Note: The key volatiles were screened out according to Spearman correlation analysis (p<0.05) and rOAV>1. Odor threshold data obtained from: a. [57]; b. [58]; n.f., not found. JK. ‘Jiukengzhong’; LJ43. ‘Longjing 43’, ZN117. ‘Zhenong 117’, and FDDB. ‘Fudingdabaicha’.

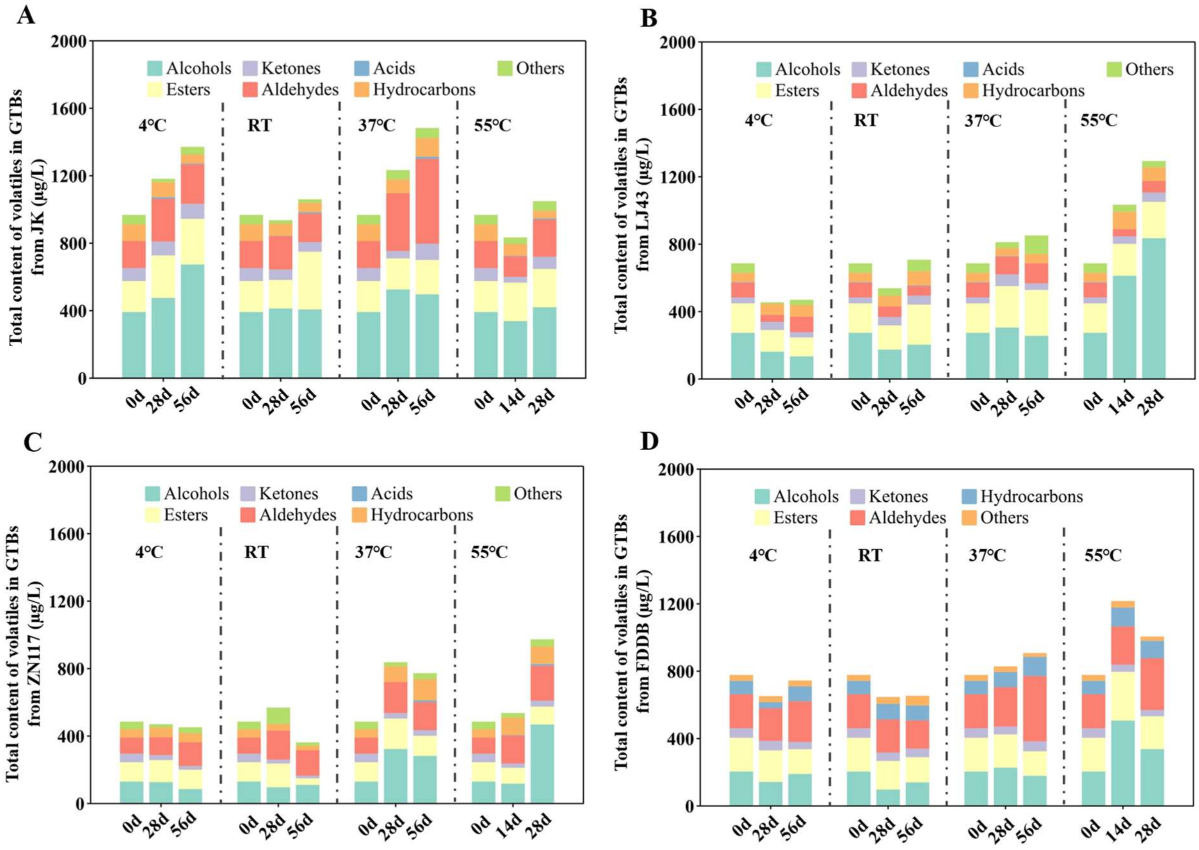

Figure S1. Changes in level of volatiles in GTBs during storage. A-B. GTBs were prepared from the shoots of cultivar 'Jiukengzhong'(JK), 'Longjing 43'(LJ43), 'Zhenong 117'(ZN117), and 'Fudingdabaicha'(FDDB), respectively.

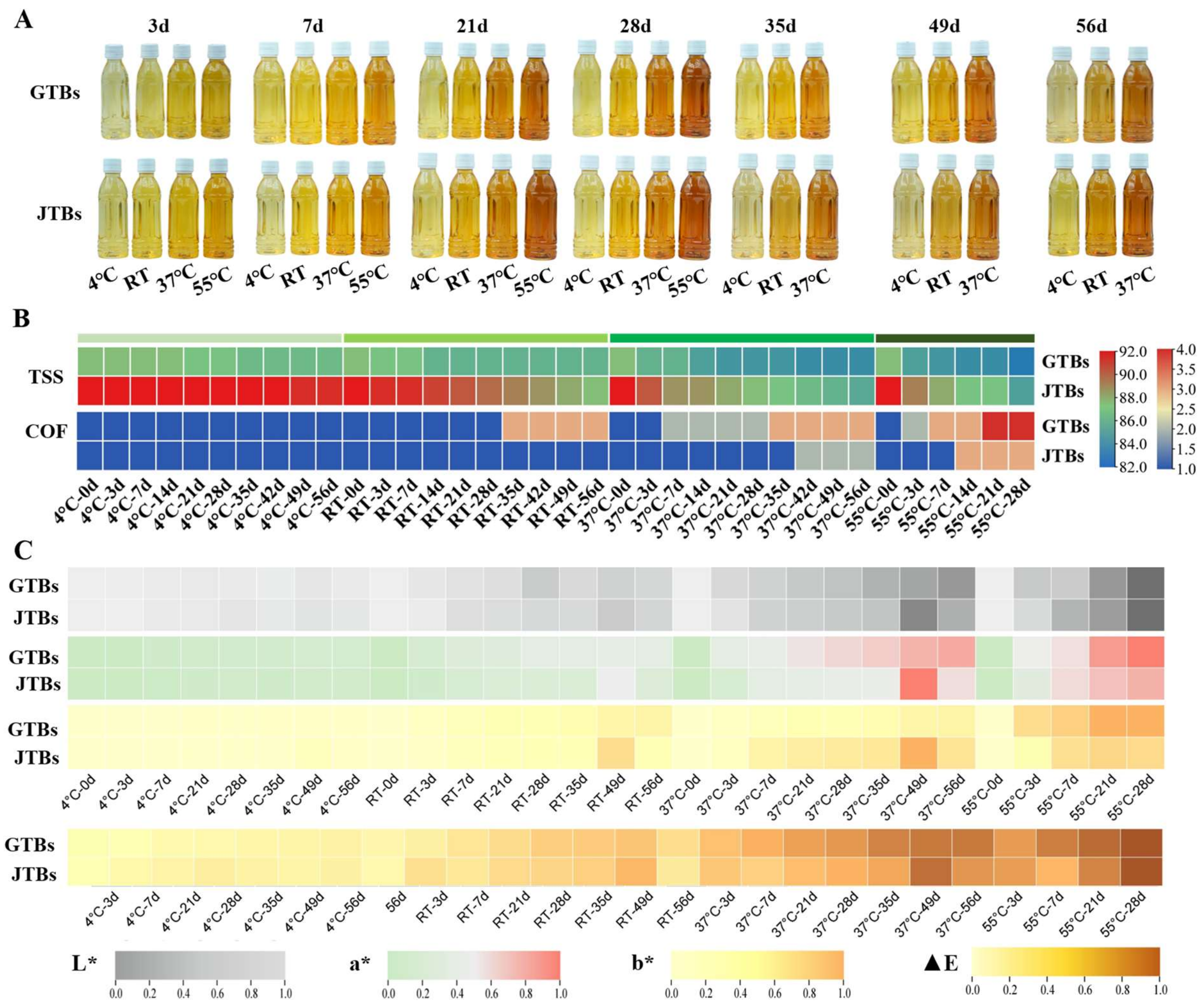

Figure S2. Changes in appearance, sensory quality, color difference of JTBs and GTBs (JGY) during storage. A. Change in appearance. A. Change in TSS and COF. C. Change in L*, a*, b* and ΔE values. GTBs. Beverages prepared from green tea of cultivar ‘Jinguanyin’ (JGY); JTBs. Beverages prepared from jasmine scented green tea of cultivar JGY. TSS. Total sensory score; COF. Cooked-off flavor. The heatmap was performed by TBtools after the data were normalized.

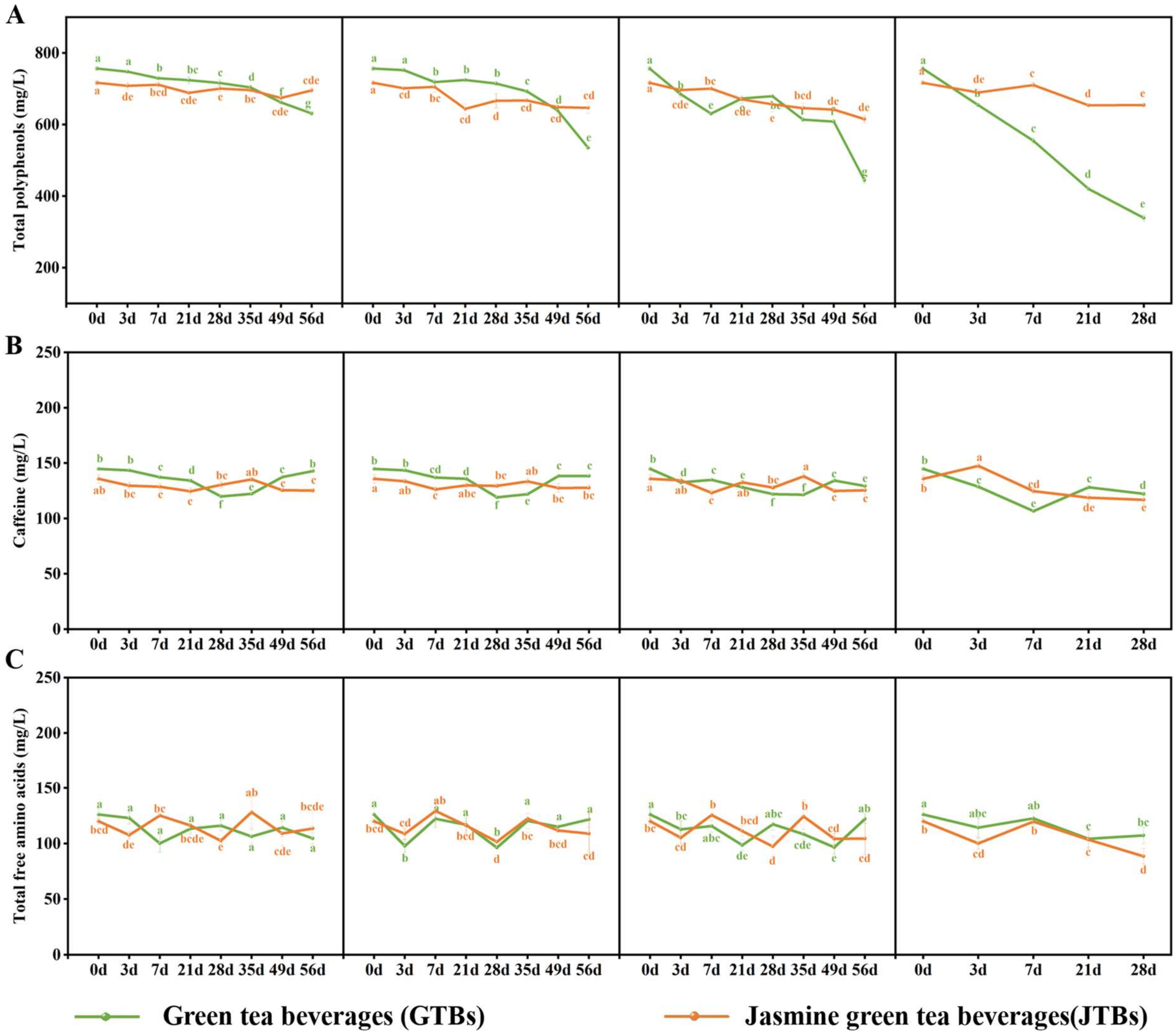

Figure S3. Dynamic changes in non-volatile components in JTBs and GTBs (JGY) during storage. A. Changes in the content of total polyphenols (TPs). B. Change in the content of caffeine. C. Change in the content of free amino acids (FAAs).

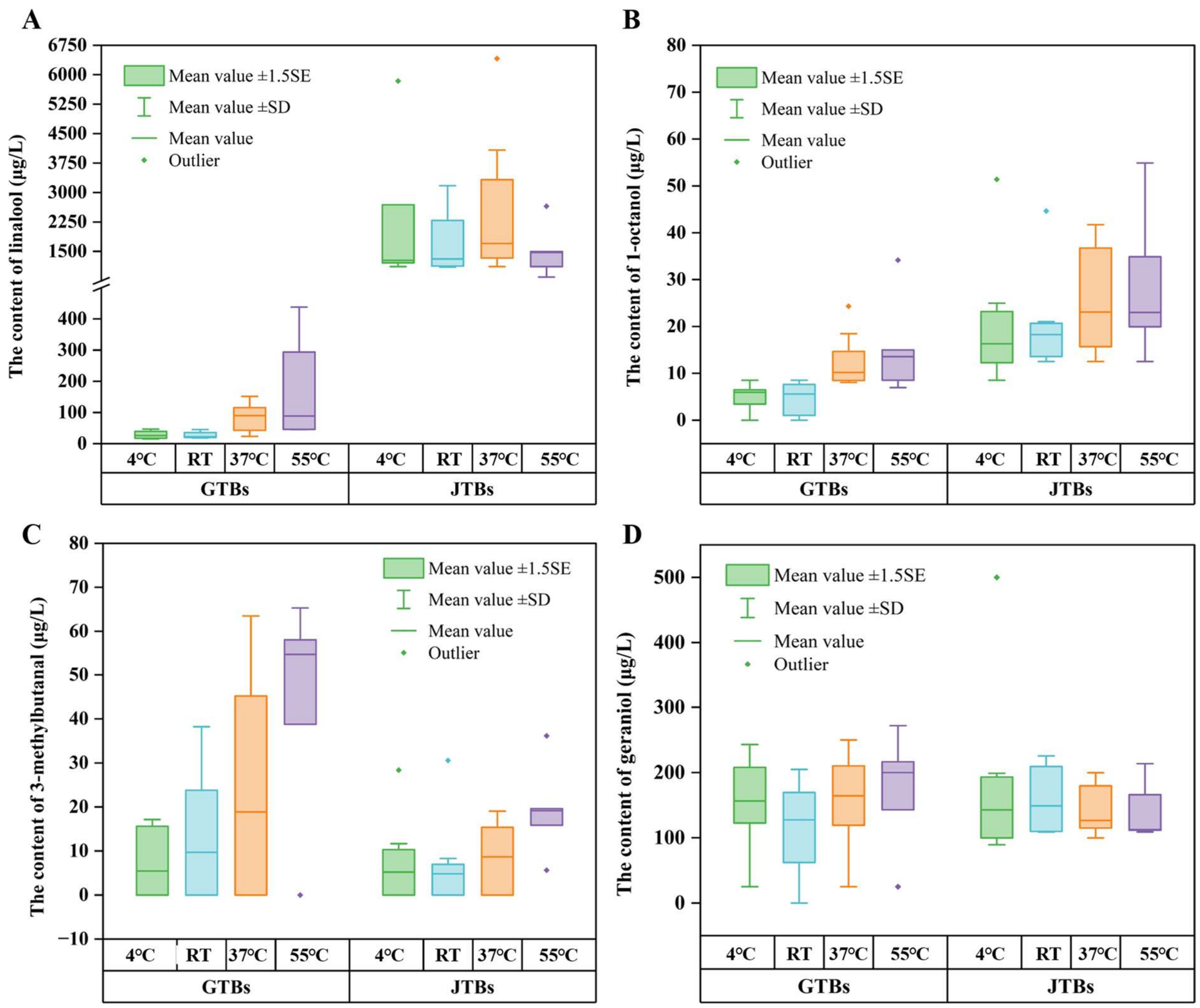

Figure S4. Change in the key volatiles related to COF in JTBs and GTBs (JGY) during storage. A. Change in the content of linalool. B. Changes in the content of 1-octanol. C. Change in the content of 3-methylbutanal. D. Change in the content of geraniol.
